# The loss of SHMT2 mediates 5-fluorouracil chemoresistance in colorectal cancer by upregulating autophagy

**DOI:** 10.1038/s41388-021-01815-4

**Published:** 2021-05-14

**Authors:** Jian Chen, Risi Na, Chao Xiao, Xiao Wang, Yupeng Wang, Dongwang Yan, Guohe Song, Xueni Liu, Jiayi Chen, Huijun Lu, Chunyan Chen, Huamei Tang, Guohong Zhuang, Guangjian Fan, Zhihai Peng

**Affiliations:** 1 Department of Thoracic Surgery, Shanghai Pulmonary Hospital, School of Medicine, Tong Ji University, Shanghai, China; 2 Department of General Surgery, Xiang An Hospital of Xiamen University, School of Medicine, Xiamen University, Xiamen, China; 3 Department of General Surgery, Hua Shan Hospital, School of Medicine, Fu Dan University, Shanghai, China; 4 Department of Gastrointestinal Surgery, The Fifth Affiliated Hospital of Sun Yat-Sen University, Zhuhai, China; 5 Department of General Surgery, Zhong Shan Hospital, School of Medicine, Fu Dan University, Shanghai, China; 6 Translational Medicine Center, Shanghai General Hospital, School of Medicine, Shanghai Jiao Tong University, Shanghai, China; 7 Department of Pathology, Guigang City People’s Hospital, Guigang, Guangxi China; 8 Department of Pathology, Shanghai Jiao Tong University Affiliated Sixth People’s Hospital, Shanghai, China; 9 Organ Transplantation Institute of Xiamen University, Fujian Provincial Key Laboratory of Organ and Tissue Regeneration, School of Medicine, Xiamen University, Xiamen, China

**Keywords:** Colorectal cancer, Autophagy

## Abstract

5-Fluorouracil (5-FU)-based chemotherapy is the first-line treatment for colorectal cancer (CRC) but is hampered by chemoresistance. Despite its impact on patient survival, the mechanism underlying chemoresistance against 5-FU remains poorly understood. Here, we identified serine hydroxymethyltransferase-2 (SHMT2) as a critical regulator of 5-FU chemoresistance in CRC. SHMT2 inhibits autophagy by binding cytosolic p53 instead of metabolism. SHMT2 prevents cytosolic p53 degradation by inhibiting the binding of p53 and HDM2. Under 5-FU treatment, SHMT2 depletion promotes autophagy and inhibits apoptosis. Autophagy inhibitors decrease low SHMT2-induced 5-FU resistance in vitro and in vivo. Finally, the lethality of 5-FU treatment to CRC cells was enhanced by treatment with the autophagy inhibitor chloroquine in patient-derived and CRC cell xenograft models. Taken together, our findings indicate that autophagy induced by low SHMT2 levels mediates 5-FU resistance in CRC. These results reveal the SHMT2–p53 interaction as a novel therapeutic target and provide a potential opportunity to reduce chemoresistance.

## Introduction

Colorectal cancer (CRC) is the third leading cause of cancer mortality worldwide due to its metastatic properties and resistance to current treatments [1]. 5-Fluorouracil (5-FU)-based adjuvant chemotherapy is a widely accepted systemic therapeutic option for CRC patients; however, there is an urgent need to better understand the underlying chemoresistance mechanism of CRC to this treatment and identify tumor cell-specific therapeutic targets for drug discovery or “repositioning” of known therapies [2, 3].

Autophagy, a catabolic process, is thought to buffer metabolic stress, thereby promoting cell survival [4, 5]. In the context of cancer, autophagy plays a puzzling role, serving as a tumor suppressor during the initial stages but later protecting tumor cells from chemo- and radioresistance, hypoxia and the immune defense system [6–11]. Various cellular stressors, including the tumor suppressor p53, can stimulate autophagy [4]. On the other hand, pharmacological interference and the knockout or knockdown of p53 can also induce autophagy [12, 13]. Therefore, p53 has a dual effect on autophagy. Cytosolic but not nuclear p53 is responsible for inhibiting autophagy [12, 13]. However, despite the critical role of cytosolic p53 in autophagy, the upstream signals controlling this protein remain unknown. In addition, the mechanism by which cytosolic p53 influences chemoresistance through autophagy requires exploration.

Because of the rapid proliferation of CRC cells, moderate serine amounts must be converted to glycine to support nucleotide biosynthesis and proliferation [14–19]. Serine hydroxymethyltransferase 2 (SHMT2) plays a regulatory role in the conversion of serine to glycine [20, 21]. SHMT2 is upregulated in cancers to support tumor cell proliferation [22–25]. It is required for glioma cell survival and renders these cells sensitive to inhibition of the glycine cleavage system [18]. SHMT2 is, therefore, a potential oncogene promoting colorectal carcinogenesis [23, 26]. SHMT2 can be deacetylated by SIRT3 and SIRT5 at Lys 95 and 280, respectively, increasing enzymatic activity and driving cancer cell proliferation [26, 27]. In addition, SHMT2 functions as a component of the BRISC-SHMT2 complex to deubiquitinate type 1 interferon (IFN) receptor chain 1 (IFNAR1) and HIV-1 Tat in the cytoplasm [28, 29]. Thus, SHMT2 not only functions as a methyltransferase but also plays a role in protein degradation, implying that its role in oncotherapy is complicated and requires further investigation.

In this study, we found via Gene Expression Omnibus (GEO) and TCGA database analysis that SHMT2 is tightly related to CRC progression [30, 31]. Strikingly, CRC patient samples with lower levels of SHMT2 exhibited greater 5-FU resistance than those with higher levels of SHMT2. We further found that SHMT2 binds to cytosolic p53 and suppresses its degradation, which in turn inhibits autophagy. Consistent with this result, the autophagy inhibitor chloroquine (CQ) increased the 5-FU chemosensitivity of CRC samples with low levels of SHMT2. Our study thus reveals a new function of SHMT2 in autophagy through the maintenance of cytosolic p53 stability instead of metabolism and suggests a potential anticancer chemotherapeutic strategy.

## Materials and methods

### Patients, cohorts, and tissue microarrays

Fifty paired fresh-frozen samples of primary colorectal carcinoma (CRC) and adjacent normal colon tissues were collected from the Department of Surgery at Shanghai General Hospital, School of Medicine, Shanghai Jiaotong University. A total of 378 paraffin-embedded samples of stage II–III primary colorectal carcinoma were collected between 2003 and 2011. Tissue microarrays were constructed from these samples. All the samples were obtained during surgery. This research was approved by the Ethics Committee of Shanghai General Hospital (2016KY069), and written informed consent was obtained from all patients before enrollment in the study.

### Nude mouse xenograft models

CRC xenografts were established in 5-week-old female BALB/c nude mice purchased from the Institute of Zoology, Chinese Academy of Sciences, Shanghai. Tumor-bearing and the calculation of tumor volumes were performed as previously described [32]. Briefly, SHMT2-sh and control cells (3 × 10^6^) were injected subcutaneously into the flanks of nude mice. 5-FU (20 mg/kg/day) was injected intraperitoneally weekly for 3 weeks. CQ (10 mg/kg) was administered as a daily oral gavage. All animal procedures were conducted in accordance with the Hospital Animal Care guidelines of Shanghai General Hospital. All efforts were made to minimize animal suffering.

### Establishment of the PDX model

Fresh tissues from 4 CRC patients (two with high SHMT2 expression and two with low SHMT2 expression) undergoing surgical treatment were obtained and implanted subcutaneously into the flanks of female NOD-Prkdc^scid^ Il2rg^tm1^/Bcgen (B-NSG) mice (Biocytogen) with a 10-gauge trocar needle. Once established, solid tumor xenografts were serially passaged using the same technique. A primary tissue sample was anonymized and obtained by the Shanghai General Hospital (Shanghai, China) Institutional Review Board.

### Cell lines, plasmids, and reagents

The human cell lines HCT116, SW480 and 293 T were purchased from the American Type Culture Collection (ATCC, Manassas, VA, USA). All the cell lines were maintained in DMEM supplemented with 10% FBS (Gibco, USA) at 37°C, 95% humidity and 5% CO^2^. The SHMT2, HDM2, p53 and p53 mutant (NLS- and NES-) sequences [13] were cloned into the pCDNA 3.0 vector or the pLVX-IRES lentiviral vector by standard cloning methods. To construct mCherry–GFP–LC3 reporter, the mCherry sequence and the cDNA sequence of LC3B were into the 5ʹ and 3ʹ regions of EGFP, respectively. The sequences of the pLKO.1-shRNAs targeting SHMT2 double-stranded oligonucleotides were as follows: 5ʹCCGGACAAGTACTCGGAGGGTTATCCTCGAGGATAACCCTCCGAGTACTTGTTTTTTG (SHMT2-sh-1), 5ʹCCGGTAGGGCAAGAGCCAGGTATAGCTCGAGTAGGGCAA-GAGCCAGGTATAGTTTTTG (SHMT2-sh-2), and 5ʹCCGGGTCTGACGTCAAGCGGAT-ATCCTCGAGGATATCCGCTTGACGTCAGACTTTTTTG (SHMT2-sh-3).

The sequence of the shRNA targeting p53 was as follow: 5ʹCCGGCGGCGCACAGAGGAAGAGAATCTCGAGATTCTCTTCCTCTGTGC-GCCGTTTTTG (p53-Sh) (all target sequences are underlined). 3-MA, CQ and sodium formate (71539, Sigma) were obtained from Sigma. Antibodies specific for LC3 (ABC929, Sigma; ab48394, Abcam), SHMT2 (NBP1-80755, Novus Biologicals, USA), p62 (p0017, Sigma), p53 (DO-1, Santa Cruz; ab32389, Abcam) and actin (A1978, Sigma) were used.

### CRISPR/Cas9 knockout cell lines

The sgRNA sequences targeting SHMT2 were designed with CRISPR Designer (http://crispr.mit.edu/). The guide sequences targeting human SHMT2 were 5ʹ- CACCGCGAGTACTTGTTGTTCAGAC-3ʹ and 5ʹ-CACCGGTTGCTGTGCTGAGC-CCGAA-3ʹ.

### Immunohistochemistry

Immunohistochemistry was performed as previously described [32]. The intensity and extent of staining were evaluated independently by two pathologists blinded to the patient outcomes. The staining score was calculated by multiplying the intensity score by the extent score. The patients with CRC were stratified by the final staining score into two groups: 0–8, lower expression; 9–12, higher expression.

### Western blot analysis and immunofluorescence

Cell lysate preparation, western blot analysis, and Immunofluorescence were performed as previously described [33, 34].

### Transmission electron microscopy

Cells were fixed with 2.5% glutaraldehyde containing 0.1 mol/L sodium cacodylate and treated with 1% osmium tetroxide. After dehydration, samples were embedded in Araldite and were then cut into thin sections that were stained with uranyl acetate and lead citrate. Digital images were obtained with a Philips CM-120 transmission electron microscope at 60 kV. Autophagosomes (APs) can be identified through their contents and double bilayers with narrow electron-lucent clefts. Autolysosomes (ALs) can be identified by their partially degraded, electron-dense contents.

### Proximity ligation assay (PLA)

Cells were permeabilized and treated with primary antibodies. Duolink^®^ In Situ PLA probes and Duolink^®^ In Situ detection reagents (Cat. No: DUO92101) were obtained from Sigma-Aldrich (Munich, Germany). The PLA assay was performed according to the manufacturer’s instructions. Cells were incubated with PLA probes for 1 h. Cells were then incubated with the ligation mix for 30 min, and the Cy3 amplification mix was applied to the slides for 100 min at 37°C. The samples were mounted using Duolink^®^ In Situ Mounting Medium with DAPI.

### mCherry-GFP-LC3 reporter assay

0.5 μg of the mCherry-GFP-LC3B plasmid and 1.5 μg of pLKO.1-shRNA plasmids expressing Scramble-sh or SHMT2-sh were cotransfected into HCT116 cells. The cells were incubated in mock medium or medium containing 100 μM CQ for 8 h and were then fixed with 4% paraformaldehyde (Sigma-Aldrich). The cells were subsequently stained with DAPI, and the formation of intracellular puncta was monitored with a Leica TCS SP8 microscope.

### Sodium formate treatment and metabolic rescue experiments

Cells were treated with sodium formate (Sigma–Aldrich) at the indicated concentrations and collected as previously described after 12 h for western blotting. WT SHMT2 plasmids and plasmids carrying the sequences for the SHMT2 K95Q [26], K280Q and E98L/Y106F [35] catalytically inactive mutants were transfected into HCT116 SHMT-sh cells (clone-2) and collected as previously described after 24 h for western blotting.

### Quantification of GFP–LC3 puncta

Cellular autophagic activities were assessed by determining the formation of GFP-LC3 aggregates in HCT116 cells and were quantified by counting the percentage of cells exhibiting the accumulation of GFP-LC3 in dots or vacuoles (GFP-LC3^vac^). Puncta were counted in a minimum of 100 cells per sample in three replicates. Cells exhibiting a mostly diffusive distribution of GFP-LC3 in the cytoplasm and nucleus were considered nonautophagic, whereas cells showing several intense punctate GFP-LC3 aggregates but no nuclear GFP-LC3 were classified as autophagic. Each GFP-LC3 stained sample was evaluated by two independent investigators.

### GC–MS analysis

The GC–MS experiments were conducted as previously described [36]. In total, six biological replicates per group were subjected to GC/MS analysis. Metabolites with variable influence on projection values greater than 1.0 and *P* < 0.05 were included.

### Statistical analysis

Student’s two-tailed *t* test and GraphPad Prism were used for statistical analysis. A *P* < 0.05 was considered to indicate a significant difference.

## Results

### Analysis of CRC via high-throughput database screening reveals that SHMT2 is pivotal in CRC

Currently, the GEO (Gene Expression Omnibus) database harbors the most comprehensive datasets for the gene expression profiles of all cancer types. To identify key genes that might significantly contribute to the prognosis of CRC, we first analyzed differentially expressed genes (DEGs) between CRC and adjacent normal tissues (control) using a machine learning approach based on five algorithms (diagonal linear discriminant analysis, Bayesian CCP, nearest neighbor, nearest centroid and support vector machines). A total of 66 genes were identified using the GSE9348 datasets [37] as the training cohort, which contained 72 CRC and 12 control cases (Fig. S1A–B). These genes were further validated in another two independent cohorts (GSE44076 [38] and GSE44861 [39]) containing 154 CRC and 153 control cases (Fig. S1C–E). In addition to some well-established oncogenes (e.g., MYC and MET), SHMT2 emerged among many DEGs that might potentially contribute to prognosis. Next, we analyzed the prognostic effect of these 66 genes by a Cox regression model in 532 tissue samples with prognostic information (GSE14333 [40], GSE17536 [41], and GSE29621 [42]) and applied the random forest test to further characterize the most valuable factors that could predict CRC prognosis (Fig. S1F–G). Five core genes that contributed most to the risk score were identified as survival predictors of CRC: risk score = −0.370 × CPM − 0.122 × GUCA2B + 0.332 × MET + 0.088 × SCN9A + 0.827 × SHMT2. Among these genes, SHMT2, which encodes one of the most prominent enzymes in cancer metabolism, has been little studied in CRC, which inspired us to investigate further.

### SHMT2 interacts with cytosolic p53

**Fig. 1:**
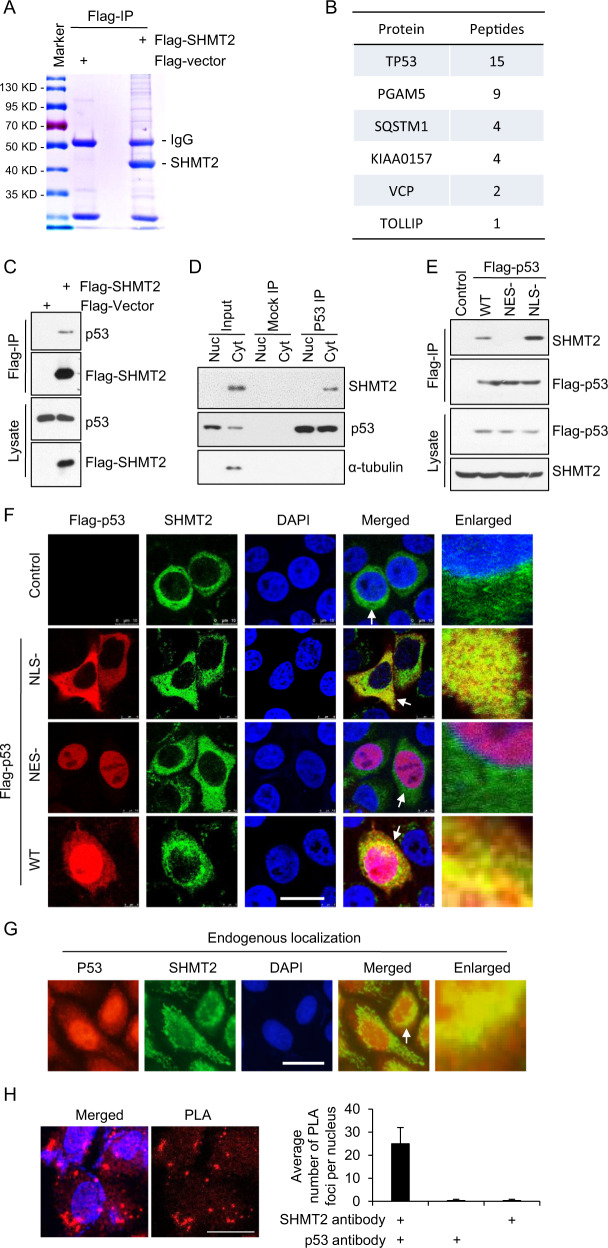
SHMT2 interacts with cytosolic p53. **A**, **B** SHMT2 purified by Flag-IP was collected after in-gel digestion and used for LC-MS/MS analysis to search for the binding proteins of SHMT2. **A** Flag-SHMT2 was transfected into 293 T cells for 24 h, isolated by coimmunoprecipitation, separated by SDS-PAGE and stained using Coomassie. **B** Tabular display of the number of tryptic peptides from each of the indicated proteins that coprecipitated with SHMT2. **C** HCT116 cells transfected with Flag-SHMT2 were immunoprecipitated with FLAG-M2 beads. Western blotting for p53 and SHMT2 was then performed. Immunoprecipitation using an anti-p53 antibody (Do-1) was followed by western blotting with anti-SHMT2 or anti-p53 antibodies (ab32389, Abcam). **D** SHMT2 interacted mainly with endogenous cytosolic p53 in HCT116 cells. *Cyt* cytosolic, *Nuc* nuclear. **E** Cytosolic p53 bound to SHMT2. HCT116 cells transfected with Flag-WT, nuclear (NES-) or cytosolic p53 (NLS-) were immunoprecipitated with FLAG-M2 beads. Western blotting for FLAG and SHMT2 was then performed. **F**–**H** Colocalization of SHMT2 and cytosolic p53. A set of partially enlarged pictures are attached on the right side. **F** Representative micrographs of HCT116 cells transfected with plasmids expressing WT, nuclear (NES-) and cytosolic p53 (NLS-). **G** Representative micrographs of HCT116 cells stained for SHMT2 and p53. **H** Representative micrographs of HCT116 cells in the proximity ligation assay (PLA). Scale bar, 10 μm. PLA foci per nucleus for the two antibodies are presented in the histogram.

Although SHMT2 is an essential enzyme in one-carbon metabolism, it is found in complex with many other important proteins, including BRISC [28, 29]. Herein, a proteomic analysis was carried out to identify potential SHMT2-interacting partners through IP/MS. As shown in Figs. 1A, B, p53 was identified as a novel SHMT2 binding protein, along with KIAA0157, a known SHMT2 binding protein (Fig. 1B). In HCT116 cells, SHMT2-Flag coimmunoprecipitated with p53 (Fig. 1C). Furthermore, we separated endogenous nuclear and cytosolic p53 and found that SHMT2 seemed to predominantly interact with the cytosolic fraction of endogenous p53 in HCT116 cells (Fig. 1D). Tumor-suppressing p53 is upregulated in response to DNA damage, oncogene activation, or exposure to other stresses [43]. Nuclear p53 acts as a transcription factor that transcriptionally activates genes involved in apoptosis and numerous other processes [43], whereas cytosolic p53 has been found to inhibit autophagy and trigger apoptosis [12, 13, 44]. As SHMT2 is localized mainly in both mitochondria and the cytoplasm [28], we constructed a wild-type (WT) p53, a cytosol-only p53 (NLS-) mutant with a disrupted nuclear localization sequence (NLS), and a nuclear-retained p53 (NES-) mutant with a disrupted nuclear export signal [13] to assess which subpopulation of cellular p53 interacted with SHMT2. The results of coimmunoprecipitation experiments showed that SHMT2 interacted with cytosolic p53 (NLS-) but not nuclear p53 (NES-) (Fig. 1E). Moreover, the immunofluorescence results showed that SHMT2 partially colocalized with cytosolic p53 in cells cotransfected with SHMT2, WT p53, nuclear p53 (NES-), or cytosolic p53 (NLS-) [13] (Fig. 1F). Consistent with the finding that p53 is also localized in the cytoplasm [44], the colocalization of endogenous SHMT2 with cytosolic p53 was also observed (Fig. 1G). A proximity ligation assay (PLA) was performed, and the data provided further evidence that these proteins colocalized with each other (Fig. 1H). Collectively, these results indicated that SHMT2 interacts with cytosolic p53.

### Depletion of SHMT2 induces autophagy

**Fig. 2:**
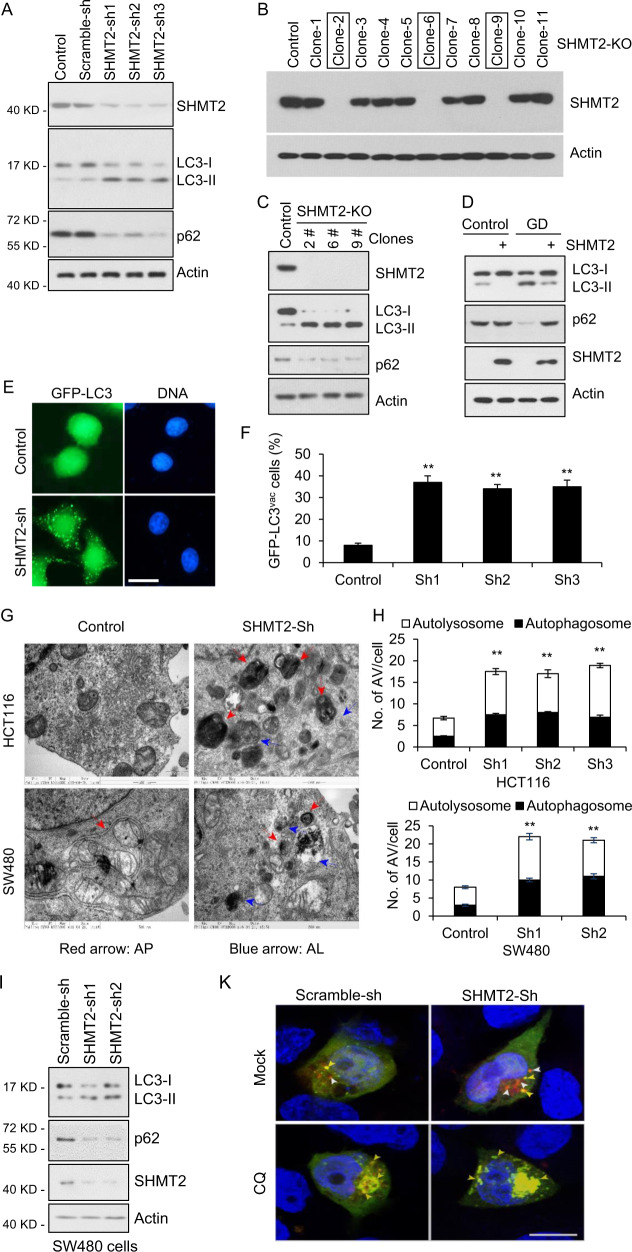

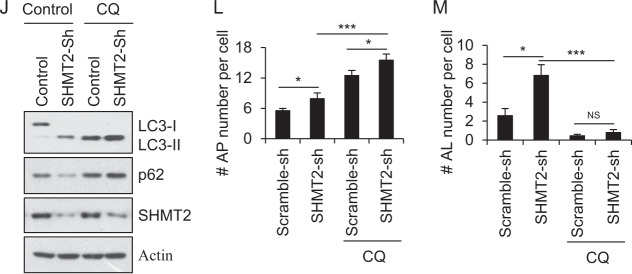
Depletion of SHMT2 induces autophagy. **A** The effect of SHMT2 on autophagy. To establish stable cell lines, HCT116 cells were infected with Scramble-sh (Control) or SHMT2 knockdown (SHMT2-sh-1, -sh-2, or -sh-3) lentivirus for 72 h and selected with puromycin (1 mg/ml). The protein levels of endogenous SHMT2, p62, LC3, and β-actin (as the internal standard) were examined by western blotting. **B** Identification of SHMT2-KO monoclonal HCT116 cell lines. **C** The protein levels of endogenous SHMT2, p62, LC3, and β-actin were evaluated in control and SHMT2-KO HCT116 cells. **D** The effect of SHMT2 on autophagy under glucose deprivation (GD) was assessed. **E** GFP-LC3 puncta were induced in SHMT2 knockdown cells. Control and SHMT2-sh stable HCT116 cell lines were transfected with the GFP-LC3 plasmid and cultured in complete medium for 24 h. Scale bar, 10 μm. **F** The percentage of cells exhibiting accumulation of GFP-LC3 in puncta (GFP-LC3^vac^) is shown (mean ± s.d., *n* = 3; **P < 0.01). **G** Ultrastructural evidence of autophagic vacuolization induced by SHMT2 depletion. **H** The numbers of autophagosomes (APs) and autolysosomes (ALs) were determined in at least 50 cells in three independent experiments (mean ± s.d.; **P < 0.01). **I** Effect of SHMT2 on LC3 maturation in SW480 cells. To establish stable cell lines, SW480 cells were infected with Scramble-sh (Control) or SHMT2 knockdown (SHMT2-sh-1 or -sh-2) lentivirus for 72 h and selected with puromycin (1 mg/ml). The protein levels of endogenous SHMT2, p62, LC3, and β-actin (as the internal standard) were examined by western blotting. **J** Autophagy levels were increased in SHMT2-sh cells and prevented by the autophagy inhibitor chloroquine (CQ). **K**–**M** Representative images and quantification of HCT116 cells expressing mCherry-GFP-LC3B and the indicated shRNA. APs and ALs were identified as yellow and red puncta, respectively. The numbers of puncta are shown as the mean ± s.e.m. values. Statistical significance was determined by Poisson regression. *ns* nonsignificant, *P < 0.05, ***P < 0.001.

Cytosolic p53 mediates the inhibition of autophagy, as deletion, depletion, or pharmacological inhibition of p53 induces autophagy in mouse, human and nematode cells [13]. Having verified the binding of SHMT2 to cytosolic p53, we then examined autophagic flux in cells with endogenous SHMT2, SHMT2 knockdown (SHMT2-sh), or SHMT2 knockout (SHMT2-KO). During AP maturation, cytoplasmic LC3 is conjugated to phosphatidylethanolamine (PE) and transported to the surface of the phagophore, increasing the ratio of PE-LC3 (LC3-II) versus cytoplasmic LC3 (LC3-I) [5, 45]. Upon autophagy activation, P62, an adaptor for autophagic substrates and a key regulator in autophagy [46], is also degraded, rendering it another commonly used reporter for cellular autophagy activity. As shown in Fig. 2A–C, the [LC3-II]/[LC3-I] ratio was markedly increased and p62 levels were decreased in SHMT2-sh and SHMT2-KO cells compared to control HCT116 cells, which clearly suggested that cellular autophagy was activated upon SHMT2 deficiency. Moreover, overexpression of SHMT2 seemed to efficiently suppress cellular autophagy triggered by glucose deprivation (Fig. 2D). To visualize the autophagic vesicles directly, we examined them by both GFP-LC3 puncta and transmission electron microscopy (TEM). Consistently, SHMT2 knockdown increased the number of LC3 puncta per cell (Fig. 2E–F). TEM analysis of autophagy in SHMT2-sh cells revealed that SHMT2 knockdown led to a marked increase in the number of autophagic vacuoles (AVs) in vitro (Fig. 2G–H). Consistent with these findings, enhanced autophagy was also observed in SW480 cells (Fig. 2I) and was inhibited by CQ (Fig. 2J). To examine autophagic flux in detail, we also monitored APs and ALs via the mCherry–GFP–LC3 reporter, which labeled APs and ALs with yellow and red fluorescence, respectively. SHMT2 knockdown increased the numbers of both APs and ALs in HCT116 cells, and CQ treatment increased the number of APs but decreased the number of ALs in SHMT2-sh cells (Fig. 2K–M). The above data indicate that autophagy is enhanced in SHMT2-low cells.

### Depletion of SHMT2 induces autophagy via degradation of cytosolic p53 in response to 5-FU treatment

Typically, SHMT2 functions to regulate one-carbon metabolism [47, 48]. We thus analyzed the metabolites in cells with or without SHMT2 knockdown and found that the metabolites that changed the most after SHMT2 knockdown were involved in arginine and proline metabolism; alanine, aspartate and dicarboxylate metabolism; pyrimidine metabolism; and valine, leucine and isoleucine biosynthesis (Fig. S2A). It has been established that cellular autophagy activities can be modulated when cell metabolism is altered [49–51]. However, as shown in Fig. S2B, treatment with sodium formate (a one-carbon metabolite mimic) did not suppress the cellular autophagy activated by SHMT2 deficiency (in SHMT2-KO cells), suggesting that the changes in one-carbon metabolites might not directly influence cellular autophagy. In other words, this result helped us decouple the enzyme activity of SHMT2 and its autophagy-suppressing function. Even stronger evidence came from the fact that enzymatically dead [26, 35] could efficiently suppress autophagy activated upon SHMT2 knockout (Fig. S2C). Altogether, we believe it is safe to conclude that the effects of SHMT2 on autophagy are not dependent on its typical function in one-carbon metabolism.

**Fig. 3:**
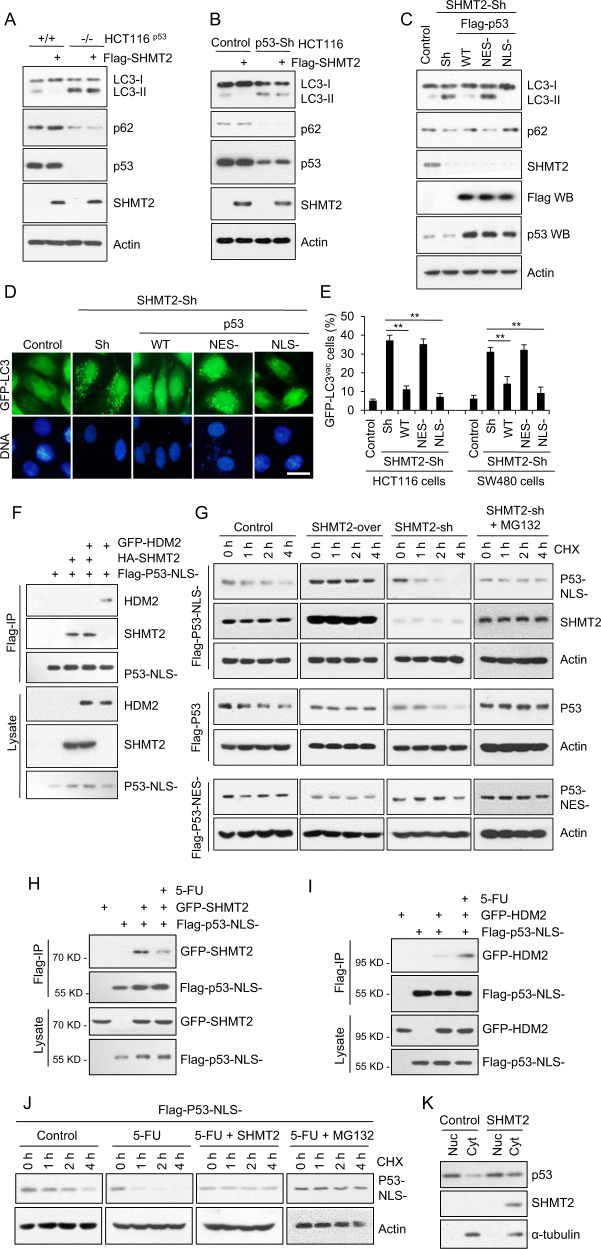
SHMT2 depletion induces autophagy via degradation of cytosolic p53 in response to 5-FU treatment. **A**–**C** Effect of SHMT2 and p53 on LC3 maturation. The protein levels of SHMT2, p53, p62, LC3, and β-actin (as the internal standard) were assessed by western blotting using anti-Flag and anti-p53, anti-p62, anti-LC3, and anti-β-actin antibodies, respectively. **A** HCT116^p53+/+^ cells and HCT116^p53-/-^ cells were transfected with Flag-SHMT2 for 24 h. **B** HCT116 cells were infected with Scramble-sh (Control) or p53 knockdown (Sh) lentivirus for 72 h and transfected with Flag-SHMT2 for 24 h. **C** Stable control and SHMT2-sh cells were transfected with Flag-WT, nuclear (NES-) and cytosolic p53 (NLS-) plasmids for 24 h. The protein levels of p53, SHMT2, p62, LC3, and β-actin were assessed by western blotting using anti-Flag and anti-p53, anti-SHMT2, anti-p62, anti-LC3, and anti-β-actin antibodies, respectively. **D** GFP-LC3 puncta formation induced by SHMT2-sh or p53 mutants. Control and SHMT2-sh stable HCT116 cell lines were transfected with Flag-WT p53, nuclear (NES-) p53, cytosolic p53 (NLS-), or GFP-LC3 plasmids and cultured in complete medium for 24 h. Scale bar, 10 μm. **E** The percentage of HCT116 and SW480 cells exhibiting accumulation of GFP-LC3 in puncta (GFP-LC3^vac^) is shown (mean ± s.d., *n* = 3; **P < 0.01). Puncta were quantified from 100 cells. **F** SHMT2 disrupted the binding of cytosolic p53 to HDM2. HCT116 cells transfected with GFP-HDM2, HA-SHMT2, and Flag-cytosolic p53 (NLS-) plasmids were immunoprecipitated with FLAG-M2 beads. Western blotting for p53, GFP, and HA was then performed. **G** SHMT2 maintained the stability of cytosolic p53. Western blot analysis of lysates of cells with stable SHMT2 overexpression and knockdown that were transfected with Flag-WT, nuclear (NES-), and cytosolic p53 (NLS-) plasmids and treated with the translation inhibitors cycloheximide (CHX, 50 μg/ml) and MG132 (25 μM, 4 h) for the indicated durations. **H**, **I** HCT116 cells transfected with Flag-cytosolic p53 (NLS-), GFP-SHMT2 or GFP-HDM2 were immunoprecipitated with FLAG-M2 beads. Western blotting for p53 and GFP was then performed. **H** 5-FU disrupted the binding of cytosolic p53 to SHMT2. **I** 5-FU promoted the binding of cytosolic p53 to HDM2. **J** Western blot analysis of lysates of HCT116 cells transfected with Flag-cytosolic p53 (NLS-) or SHMT2 plasmids and treated with CHX and MG132 for the indicated durations with or without 5-FU. **K** Nuclear-cytosolic separation shows that SHMT2 affects the stability of endogenous cytosolic p53. *Cyt* cytosolic, *Nuc* nuclear.

Next, we evaluated whether SHMT2 regulates autophagy via cytosolic p53. SHMT2 overexpression affected neither the LC3-II/I ratio nor the p62 level in the absence of p53 (Fig. 3A). Similar results were also observed in p53 knockdown cells (Fig. 3B). To further assess whether SHMT2-mediated autophagy inhibition is p53 dependent, we restored p53 expression in SHMT2-sh cells and found that WT p53 and cytosolic p53 (NLS-) but not nuclear p53 (NES-) reversed the induction of autophagy resulting from SHMT2 depletion (Fig. 3C). Similarly, WT p53 and cytosolic p53 decreased the number of LC3 puncta per cell and the level of GFP–LC3 in SHMT2-sh cells (Fig. 3D–E). Collectively, these results indicate that SHMT2 inhibits autophagy via cytosolic p53.

Induction of autophagy has been reported to stimulate proteasome-mediated degradation of p53 through a pathway dependent on the E3 ubiquitin ligase HDM2 [13, 43]. Therefore, we investigated whether SHMT2 interferes with p53–HDM2 binding. The results of a coimmunoprecipitation competition assay showed that SHMT2 prevents cytosolic p53 from interacting with HDM2 (Fig. 3F). SHMT2 stabilized cytosolic p53 but not nuclear p53; moreover, MG132 counteracted the SHMT2-sh-induced degradation of cytosolic p53 (Fig. 3G). After 5-FU treatment, SHMT2–p53 binding was decreased (Fig. 3H), and p53–HDM2 binding was increased (Fig. 3I). 5-FU markedly accelerated cytosolic p53 protein degradation, while SHMT2 overexpression and MG132 treatment delayed cytosolic p53 protein degradation (Fig. 3J). Thus, overexpression of SHMT2 promoted the protein accumulation of endogenous cytosolic p53 (Fig. 3K). Taken together, these results indicate that SHMT2 stabilizes p53 by preventing its HDM2-mediated degradation in response to 5-FU treatment.

### Inhibition of autophagy induced by low SHMT2 expression sensitizes CRC cells to 5-FU treatment

**Fig. 4:**
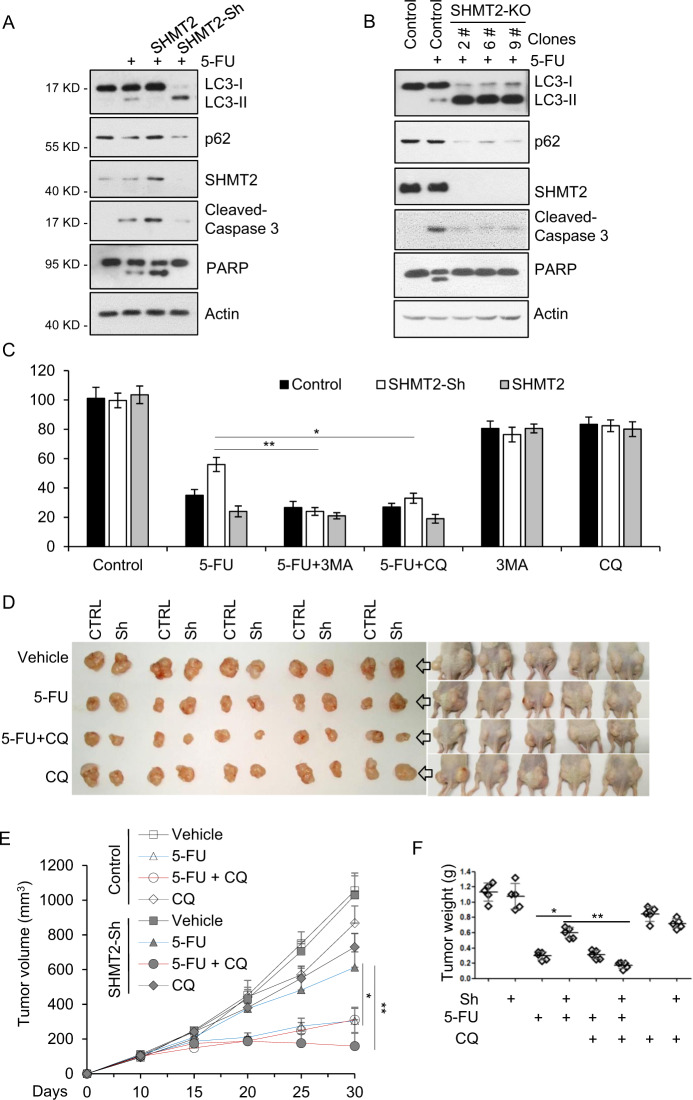
Inhibition of autophagy induced by low SHMT2 expression sensitizes CRC cells to 5-FU treatment. **A** SHMT2 promoted apoptosis and inhibited autophagy in response to 5-FU treatment. Western blot analysis of lysates of HCT116 cells that were transfected with SHMT2 or infected with SHMT2-sh lentivirus and treated with 5-FU (10 μM) for 24 h. The protein levels of SHMT2, p62, LC3, cleaved Caspase 3, PARP, and β-actin (as the internal standard) were assessed with the indicated antibodies. **B** The protein levels of SHMT2, p62, LC3, cleaved Caspase 3, PARP, and β-actin (as the internal standard) were assessed in SHMT2-KO HCT116 cells. **C** The indicated cells were treated with 5-FU (2 μM), 3-MA (10 mM) or chloroquine diphosphate salt (CQ, 20 μM) for 4 days and analyzed using the MTT cell viability assay. *P < 0.05, **P < 0.01. **D**–**F** The xenograft experiment with Control and SHMT2-sh cells treated with 5-FU or CQ is described in the Methods section. **D** Xenograft tumors were harvested and photographed. **E**, **F** Quantification of the average volumes (**E**) and weights (**F**) of the xenograft tumors are shown. Five tumors from individual mice were included in each group; *P < 0.05, **P < 0.01.

Given that cytosolic p53 triggers apoptosis and inhibits autophagy [44], we further analyzed the balance between apoptosis and autophagy in SHMT2-ov, SHMT2-sh, and SHMT2-KO cells after 5-FU treatment. In SHMT2-ov cells, the levels of cleaved caspase-3 and poly (ADP-ribose) polymerase (PARP) were increased and that of LC3-II was decreased, while the opposite pattern was observed in SHMT2-sh and SHMT2-KO cells, indicating that SHMT2 promotes apoptosis and inhibits autophagy in response to 5-FU treatment (Fig. 4A–B). Autophagy induction prevents tumor cells from undergoing apoptosis and subsequently leads to chemoresistance [10]. The autophagy inhibitors 3-methyladenine (3-MA) and CQ were then used to sensitize cells to 5-FU–based chemotherapy [9, 52]. Compared with control and SHMT2-ov cells, SHMT2-sh cells showed 5-FU resistance, which was counteracted by 3-MA and CQ (Fig. 4C).

Next, we investigated whether CQ increases sensitivity to 5-FU treatment in SHMT2-sh xenograft tumors. Control and SHMT2-sh HCT116 cells were injected subcutaneously into nude mice above the left and right hind legs, respectively. Tumor growth was markedly inhibited in SHMT2-sh–injected mice after CQ and 5-FU treatment compared with CQ treatment alone (Fig. 4D–F). 5-FU treatment and the combined treatment of CQ and 5-FU caused an ~15% reduction in the body mass of nude mice, while CQ treatment alone caused a 10% reduction in body mass, indicating that the combined therapy did not further potentiate this marker of host toxicity (Fig. S3). Collectively, our findings show that autophagy induced by low SHMT2 expression leads to 5-FU resistance and that inhibition of autophagy sensitizes SHMT2-low CRC cells to 5-FU treatment.

### 5-FU resistance is related to low SHMT2 expression and autophagy in human CRC

**Fig. 5:**
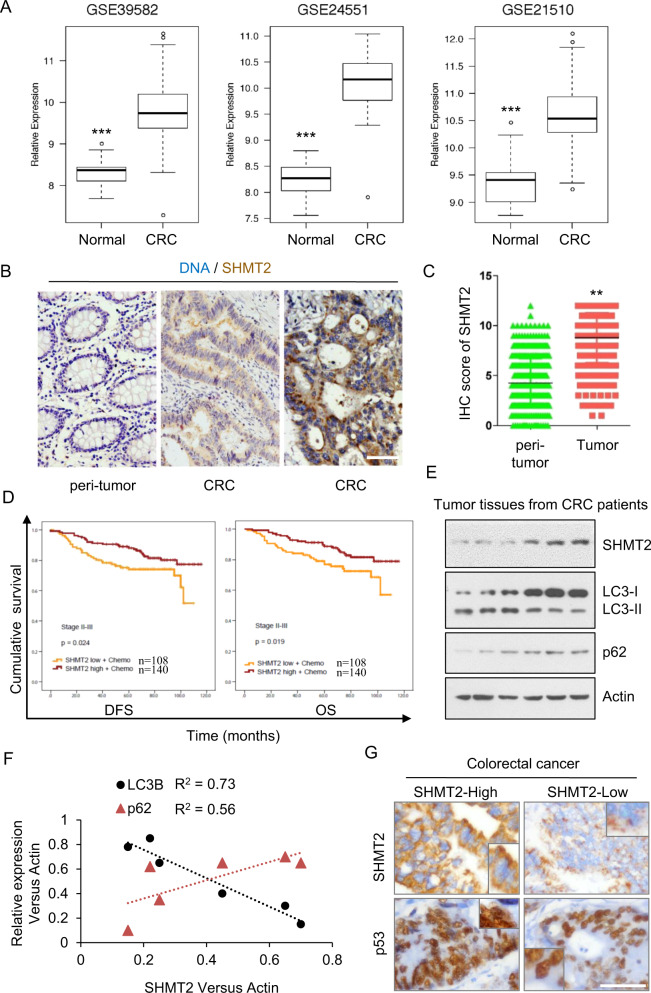
5-FU resistance is related to low SHMT2 expression and autophagy in CRC. **A** Expression of SHMT2 in three GEO datasets (GSE39582, GSE24551, and GSE21510). ***P < 0.001. **B** Representative images of immunohistochemical staining for SHMT2 in peritumor and CRC tissues. Scale bar, 50 μm. **C** 378 stage II–III paired CRC tissues assessed by immunohistochemistry are shown. **P < 0.01. **D** Survival of patients stratified by the SHMT2 expression level. DFS and OS of patients with stage II–III disease treated with 5-FU-based chemotherapy stratified by the SHMT2 expression level. **E**, **F** The protein levels of endogenous SHMT2, p62, LC3, and β-actin (as the internal standard) were examined by western blotting in CRC tissues. **F** The Spearman rank correlation test was used to evaluate correlations between the SHMT2, p62, and LC3 expression status in CRC tissues as determined by western blotting. **G** Representative images of immunohistochemical staining. Scale bar, 50 μm.

SHMT2 is a potential cancer driver gene and promotes colorectal carcinogenesis [23, 26]; moreover, it is related to 5-FU resistance in CRC cells and xenograft tumors. Thus, we further studied the role of SHMT2 in CRC therapy. q-PCR analysis of 50 paired CRC tissues and adjacent normal tissues showed that SHMT2 expression was significantly upregulated in CRC tissues compared with normal tissues (Fig. S4A). Moreover, we retrieved SHMT2 mRNA expression data from the GEO and TCGA databases and found that the expression level of SHMT2 was significantly higher in CRC tissues than in normal mucosa (Fig. S4B and Fig. 5A).

Next, we selected CRC patients with TNM stage II or III disease (*n* = 378) to explore the function of SHMT2 in response to 5-FU–based adjuvant chemotherapy (Table S1). Immunohistochemical (IHC) staining revealed higher expression of SHMT2 in human CRC specimens than in normal specimens (Fig. 5B–C). However, consistent with the complexity of colorectal tumorigenesis, 43.39% (*n* = 164) of the CRC tissues exhibited low SHMT2 expression (Table S1). To more thoroughly understand the contribution of SHMT2 to the prognosis of patients with CRC, especially its effect on the response to 5-FU–based adjuvant chemotherapy, we investigated the correlation of SHMT2 expression levels with disease-free survival (DFS) and overall survival (OS) in CRC patients. Surprisingly, patients with SHMT2-low CRC (SHMT2-low+chemo, *n* = 108; SHMT2-high+chemo, *n* = 140) treated with 5-FU–based adjuvant chemotherapy had worse DFS and OS than those with SHMT2-high CRC (Fig. 5D). Moreover, correlation analysis revealed significant correlations between the protein expression levels of SHMT2 and both LC3-II and p62, indicating that autophagy was induced in SHMT2-low CRC cells (Fig. 5E–F). Moreover, cytosolic p53 was almost undetectable in tissues with low SHMT2 expression (Fig. 5G). Given that 43.39% of the patients had SHMT2-low CRC, we need to explore the mechanism underlying 5-FU resistance. These results also imply that SHMT2 expression could be used as a therapeutic marker for clinical 5-FU resistance.

Taken together, these results further demonstrate that SHMT2 upregulation not only promotes CRC progression but also plays a vital role in mediating 5-FU-based chemoresistance in CRC patients. Furthermore, multivariate Cox proportional hazards analysis suggests that SHMT2 is a new independent marker for the prognosis of CRC patients treated with 5-FU-based chemotherapy (Tables S2, S3).

### CQ sensitizes patient-derived xenografts (PDXs) with low SHMT2 expression to 5-FU treatment

**Fig. 6:**
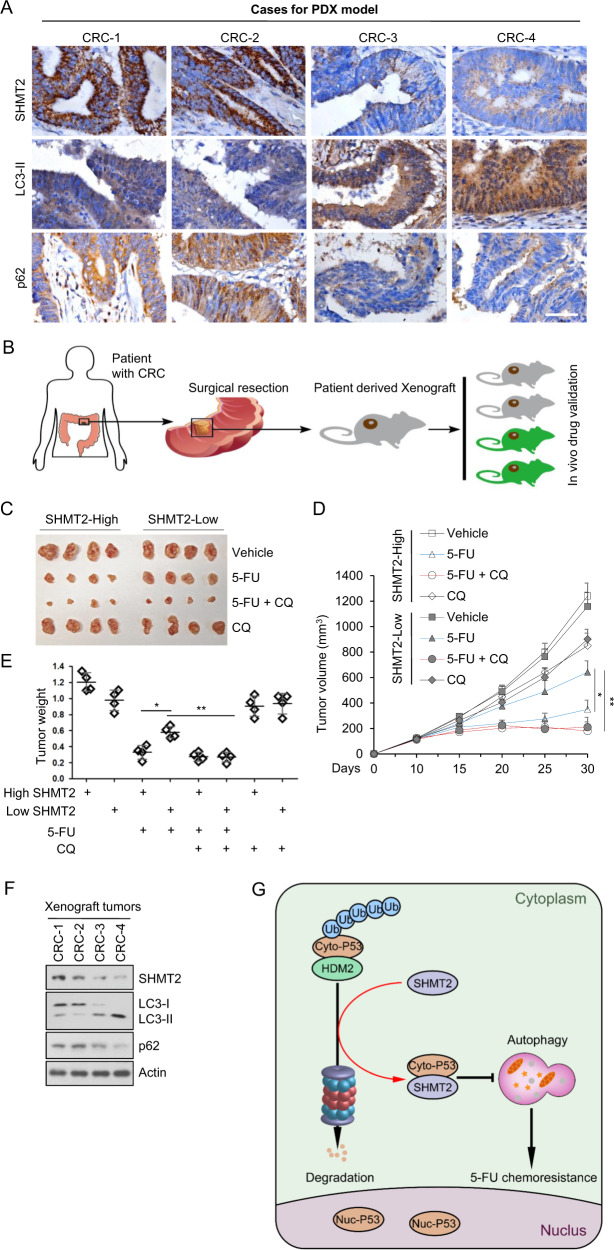
CQ sensitizes PDXs with low SHMT2 expression to 5-FU treatment. **A** Images of immunohistochemical staining for SHMT2, LC3, and p62 in CRC tissues from four selected patients (two with low SHMT2 expression and two with high SHMT2 expression) using the indicated antibodies. Scale bar, 50 μm. **B** Schematic of PDX model establishment. **C**–**E** Xenograft experiments with 5-FU or CQ treatment are described in the Methods section. **C** Xenograft tumors were harvested and photographed. **D**, **E** Quantification of the average volumes (**D**) and weights (**E**) of the xenograft tumors are shown. Four tumors from individual mice were included in each group; *P < 0.05, **P < 0.01. **F** Representative western blot of xenograft tumors. **G** Schematic diagram showing the basic hypothesis/conclusion/model.

The above results showed that low SHMT2 induced 5-FU resistance through autophagy activation. To explore the function of autophagy inhibitors in 5-FU therapy, we established a xenograft mouse model in which four CRC patient-derived tissues (two with high expression of SHMT2 and two with low expression of SHMT2) were implanted subcutaneously (Fig. 6A–B). A similar result on autophagy levels was observed in xenograft tumors and the abovementioned experiments (Fig. 6A). As shown in Fig. 6C, tumor growth was markedly inhibited in mice bearing SHMT2-low xenografts that received combination therapy with CQ and 5-FU compared to their counterparts receiving 5-FU monotherapy (Fig. 6C–E). These results indicate that the combination of CQ and 5-FU markedly inhibited tumor growth in mice bearing SHMT2-low tumors. The expression of SHMT2 is negatively related to autophagy (Fig. 6F) and is not altered in tumors during drug treatment (Fig. S5). Collectively, these findings show that CQ, as an autophagy inhibitor, sensitizes xenografts with low SHMT2 expression to 5-FU treatment (Fig. 6G).

## Discussion

We screened 66 differentially expressed genes associated with CRC progression in 224 colon cancer tissues and 165 adjacent normal tissues from three GEO data sets and found that SHMT2 is important in CRC metabolism. SHMT2, responsible for the conversion of serine to glycine, supports cancer cell proliferation in various cancers [18, 21, 53]. SHMT2 is upregulated in CRC and plays a vital role in colorectal carcinogenesis [23, 27]. Consistent with the genetic diversity of tumors, 43.39% of CRC tumors were found to have low expression of SHMT2. However, patients with SHMT2-low CRC tumors exhibited 5-FU chemoresistance and poor prognosis. Further analysis revealed that low SHMT2 induced autophagy and subsequently triggered 5-FU resistance. Via MS, we identified cytosolic p53 as a SHMT2 binding protein and found that SHMT2 inhibited autophagy by stabilizing cytosolic p53. Depletion of SHMT2 promoted autophagy and inhibited apoptosis after 5-FU treatment. Inhibition of autophagy induced by low SHMT2 expression sensitized CRC cells to 5-FU treatment in vivo and in vitro. Finally, we enhanced the lethality of 5-FU to CRC cells through treatment with the autophagy inhibitor CQ in a PDX model. These findings are essential for understanding the response to 5-FU chemotherapy in patients with SHMT2-low CRC.

Autophagy plays opposing and context-dependent roles in cancer, and the therapeutic targeting of autophagy in cancer is sometimes viewed as controversial [6]. In our study, we found that low expression of SHMT2 increased the resistance of CRC cells to 5-FU treatment through autophagy induction. The clinical data also verified this finding. In vivo and in vitro depletion of SHMT2 induced 5-FU resistance, while treatment with autophagy inhibitors decreased this resistance. Thus, our study supports the hypothesis that autophagy inhibitors are beneficial to the response to 5-FU–based chemotherapy in CRC.

The factors inducing autophagy are complex (for example, starvation, treatment with rapamycin and exposure to toxins affecting the endoplasmic reticulum) [54, 55]. Inhibition of p53 led to autophagy, and cytosolic p53 repressed the enhancement of autophagy in p53^–/–^ cells. Some inducers of autophagy stimulate proteasome-mediated degradation of p53 via the E3 ubiquitin ligase HDM2. However, the factors regulating the binding of HDM2 to p53 require exploration. Here, we found that SHMT2 competitively bound to cytosolic p53 to exclude HDM2 and thus inhibited autophagy. Treatment with 5-FU increased the binding of p53 to HDM2 to induce autophagy but decreased the binding of cytosolic p53 to SHMT2. In summary, we verified a new autophagy regulation mechanism involving the SHMT2–p53–HDM2 competitive binding system and confirmed the importance of this mechanism in mediating the response to CRC 5-FU–based chemotherapy. Indeed, it was unlikely that the p53 dependent role of SHMT2 in autophagy regulation and its sensitizing effect in 5-FU treatment was only limited to CRC. Further study is warranted to determine whether this mechanism holds true in other tumor types.

SHMT2 is located not only in mitochondria but also in the cytoplasm, as shown by our data and data from other studies [28]. SHMT2 is a tetrameric metabolic enzyme involved in one-carbon metabolism and can also participate in the BRISC-SHMT complex to deubiquitinate IFNAR1 and regulate interferon responses [28]. Here, we found that SHMT2 regulated autophagy not by controlling one-carbon metabolism but by binding to cytosolic p53. These findings emphasize the functional multiformity of SHMT2 and improve the overall understanding of the function of SHMT2 in one-carbon metabolism and autophagy.

## Conclusion

Given the complexity of tumorigenesis, mechanisms affecting chemotherapy require further exploration. Our study showed that low expression of SHMT2 is related to 5-FU resistance in CRC, implying that SHMT2 expression could be used as a therapeutic marker for clinical 5-FU resistance. Investigation of the molecular mechanism showed that SHMT2 competitively binds to cytosolic p53 to exclude the E3 ubiquitin ligase HDM2 and SHMT2 depletion decreases the stability of cytosolic p53 to induce autophagy, which maintains the survival of cancer cells treated with 5-FU. These findings reveal the SHMT2–p53 interaction as a novel oncotherapeutic target and provide a potential opportunity to reduce 5-FU resistance using autophagy inhibitors in chemotherapy.

## Supplementary information

Supplemental Figures

Supplemental Table
