# Supplementary material for: The loss of SHMT2 mediates 5-fluorouracil chemoresistance in colorectal cancer by upregulating autophagy
Source: Oncogene. 2021 May 14;40(23):3974–88. doi: 10.1038/s41388-021-01815-4 (PMC8195740; doi:10.1038/s41388-021-01815-4)
Supplement: Supplementary file 1 — Supplemental Figures [file 41388_2021_1815_MOESM1_ESM.pptx]

## Slide 1
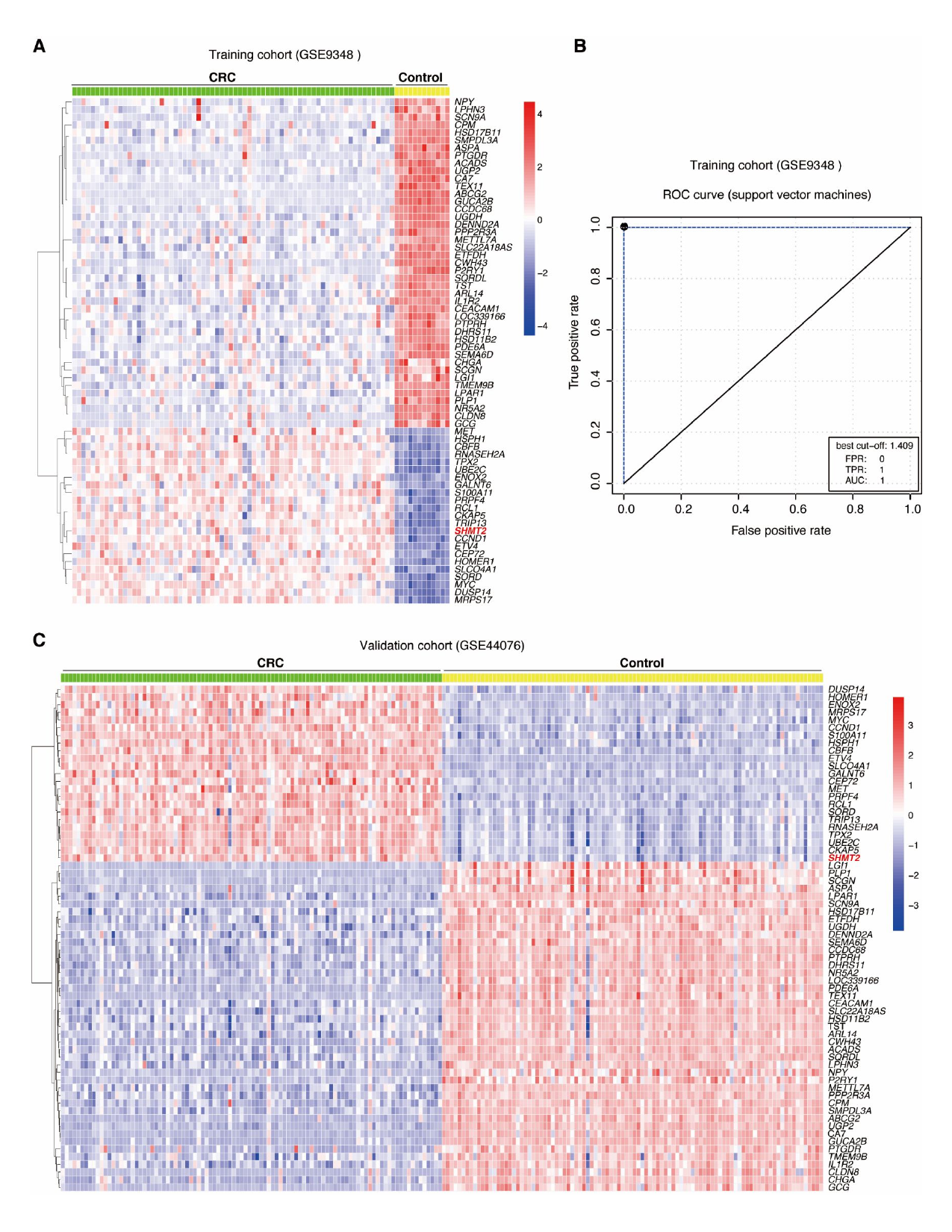

## Slide 2
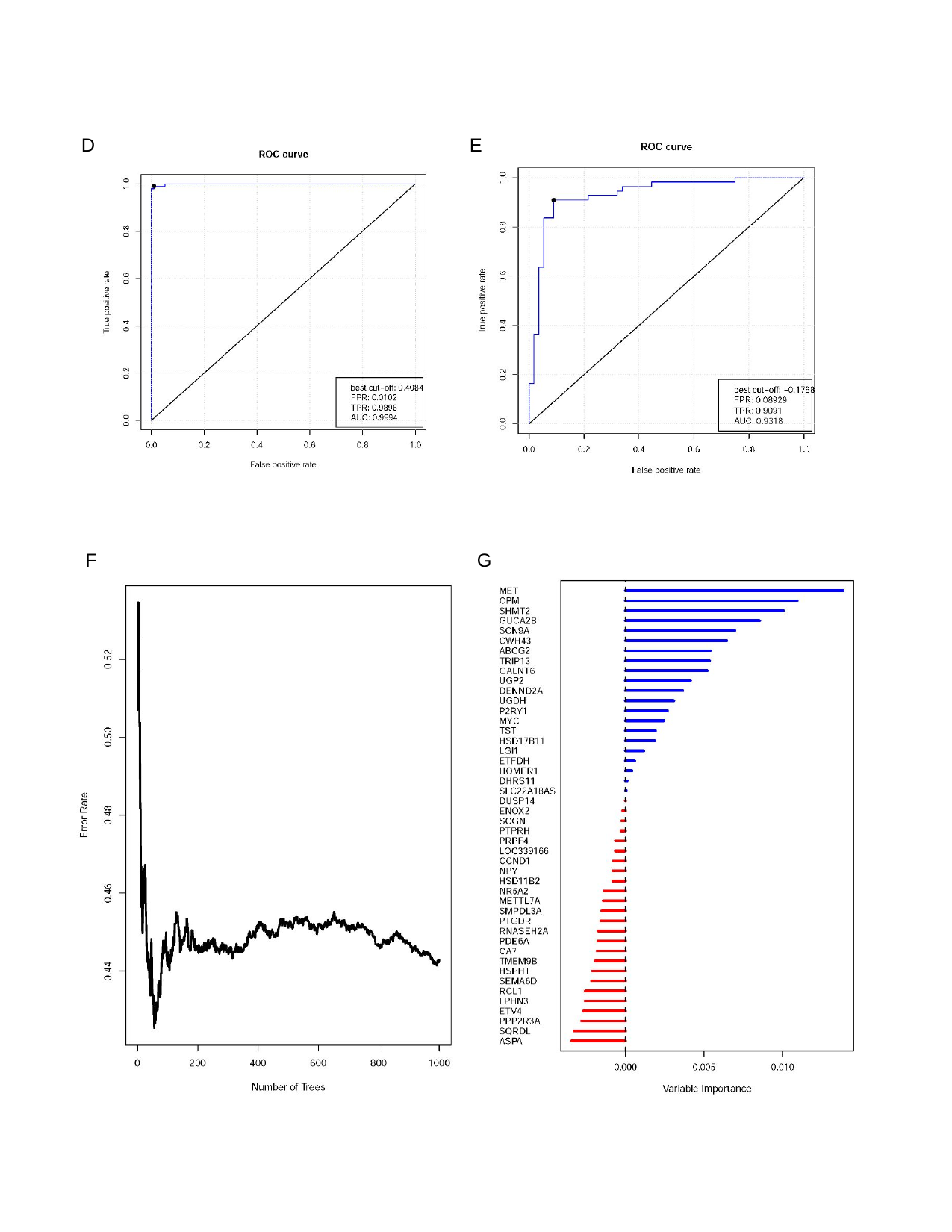

D
E
F
G

## Slide 3
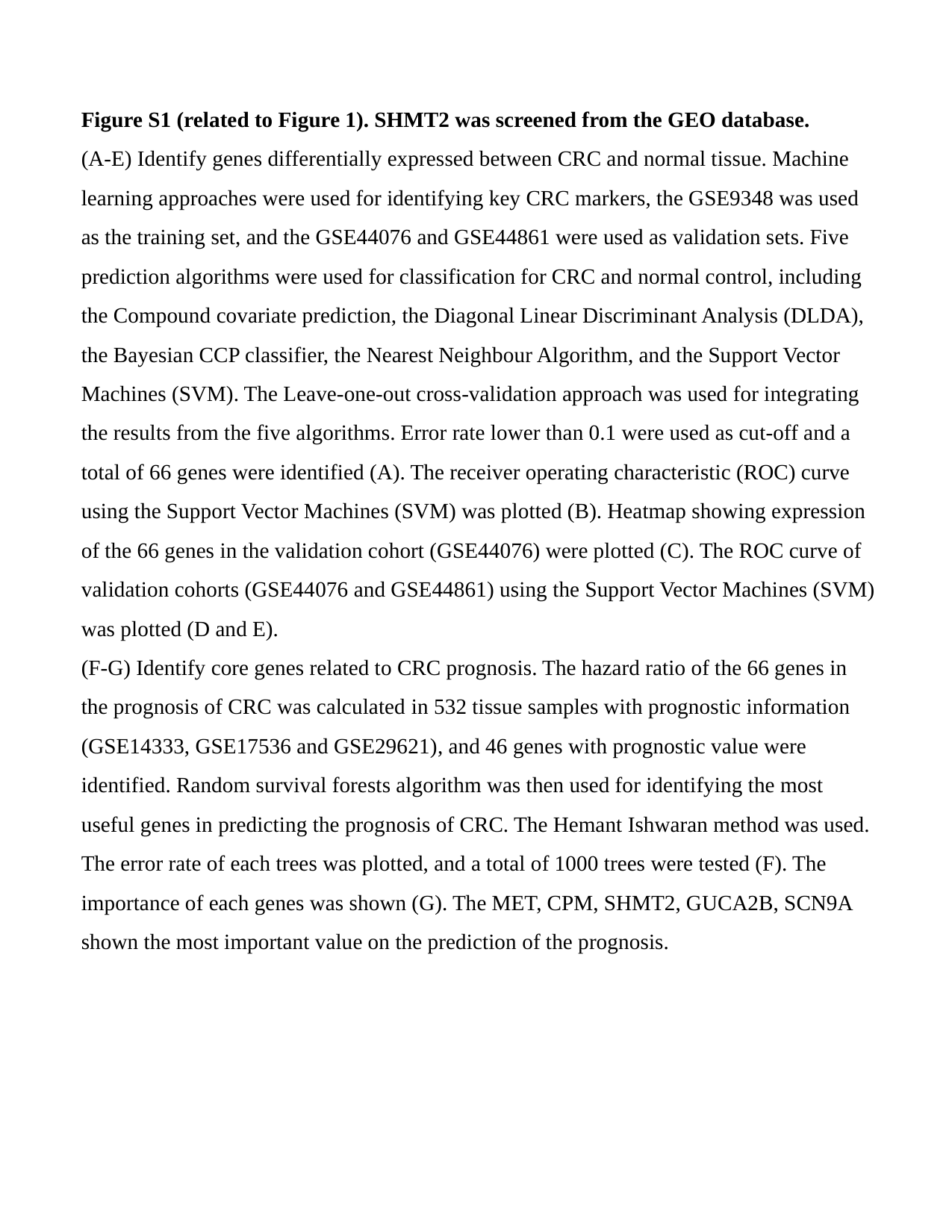

Figure S1 (related to Figure 1). SHMT2 was screened from the GEO database.
(A-E) Identify genes differentially expressed between CRC and normal tissue. Machine learning approaches were used for identifying key CRC markers, the GSE9348 was used as the training set, and the GSE44076 and GSE44861 were used as validation sets. Five prediction algorithms were used for classification for CRC and normal control, including the Compound covariate prediction, the Diagonal Linear Discriminant Analysis (DLDA), the Bayesian CCP classifier, the Nearest Neighbour Algorithm, and the Support Vector Machines (SVM). The Leave-one-out cross-validation approach was used for integrating the results from the five algorithms. Error rate lower than 0.1 were used as cut-off and a total of 66 genes were identified (A). The receiver operating characteristic (ROC) curve using the Support Vector Machines (SVM) was plotted (B). Heatmap showing expression of the 66 genes in the validation cohort (GSE44076) were plotted (C). The ROC curve of validation cohorts (GSE44076 and GSE44861) using the Support Vector Machines (SVM) was plotted (D and E).
(F-G) Identify core genes related to CRC prognosis. The hazard ratio of the 66 genes in the prognosis of CRC was calculated in 532 tissue samples with prognostic information (GSE14333, GSE17536 and GSE29621), and 46 genes with prognostic value were identified. Random survival forests algorithm was then used for identifying the most useful genes in predicting the prognosis of CRC. The Hemant Ishwaran method was used. The error rate of each trees was plotted, and a total of 1000 trees were tested (F). The importance of each genes was shown (G). The MET, CPM, SHMT2, GUCA2B, SCN9A shown the most important value on the prediction of the prognosis.

## Slide 4
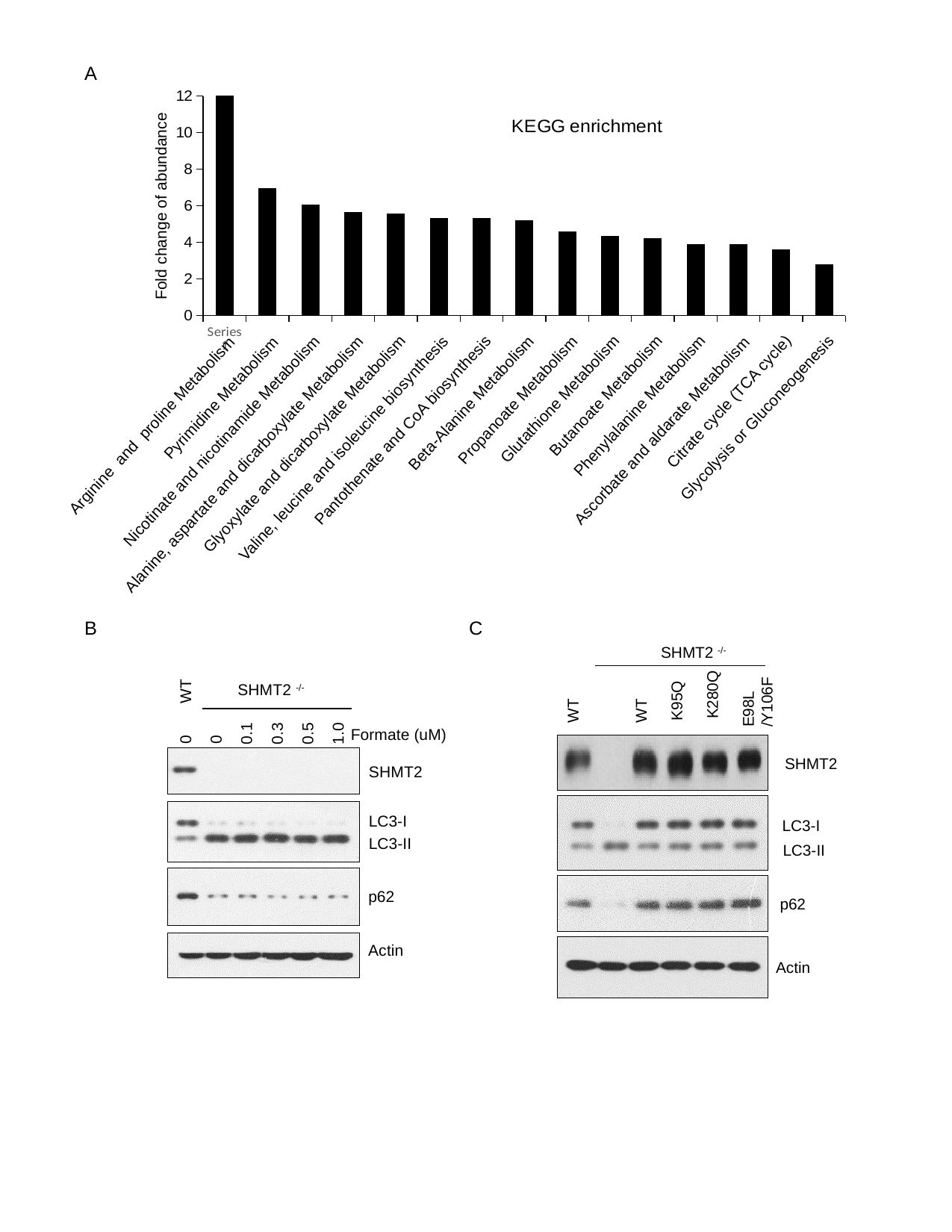

A
### Chart:
| Category | KEGG enrichment |
|---|---|
| | 12.356 |
| | 6.9315 |
| | 6.0294 |
| | 5.6337 |
| | 5.5569 |
| | 5.2926 |
| | 5.2926 |
| | 5.1883 |
| | 4.5593 |
| | 4.3325 |
| | 4.1924 |
| | 3.8753 |
| | 3.8753 |
| | 3.5745 |
| | 2.7697 |Butanoate Metabolism
Propanoate Metabolism
Pyrimidine Metabolism
Phenylalanine Metabolism
Citrate cycle (TCA cycle)
Beta-Alanine Metabolism
Glutathione Metabolism
Glycolysis or Gluconeogenesis
Arginine and proline Metabolism
Ascorbate and aldarate Metabolism
Pantothenate and CoA biosynthesis
Nicotinate and nicotinamide Metabolism
Glyoxylate and dicarboxylate Metabolism
Valine, leucine and isoleucine biosynthesis
Alanine, aspartate and dicarboxylate Metabolism
Fold change of abundance
B
C
SHMT2 -/-
E98L /Y106F
K280Q
K95Q
WT
WT
SHMT2
LC3-I
LC3-II
p62
Actin
SHMT2 -/-
WT
1.0
0.1
0.3
0.5
Formate (uM)
0
0
SHMT2
LC3-I
LC3-II
p62
Actin

## Slide 5
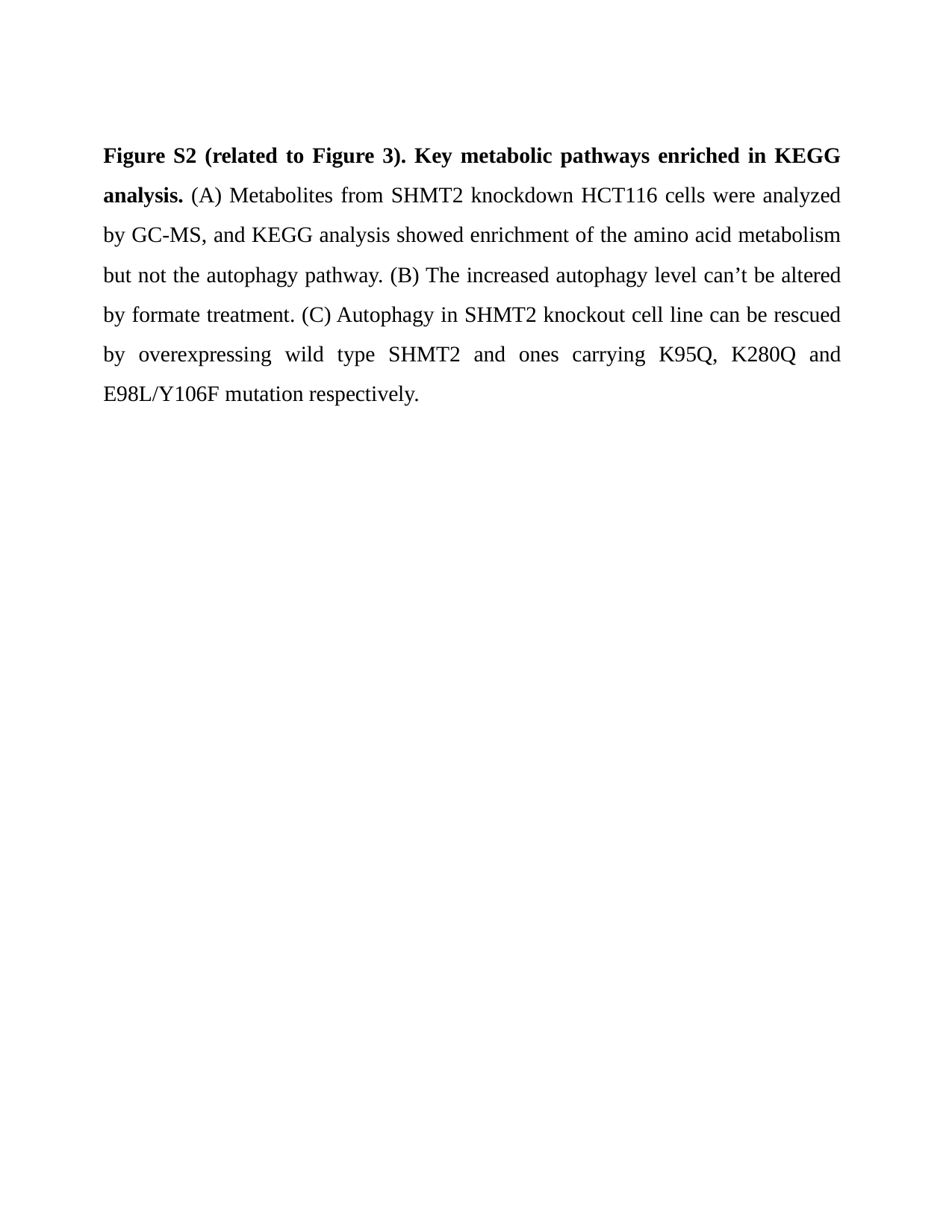

Figure S2 (related to Figure 3). Key metabolic pathways enriched in KEGG analysis. (A) Metabolites from SHMT2 knockdown HCT116 cells were analyzed by GC-MS, and KEGG analysis showed enrichment of the amino acid metabolism but not the autophagy pathway. (B) The increased autophagy level can’t be altered by formate treatment. (C) Autophagy in SHMT2 knockout cell line can be rescued by overexpressing wild type SHMT2 and ones carrying K95Q, K280Q and E98L/Y106F mutation respectively.

## Slide 6
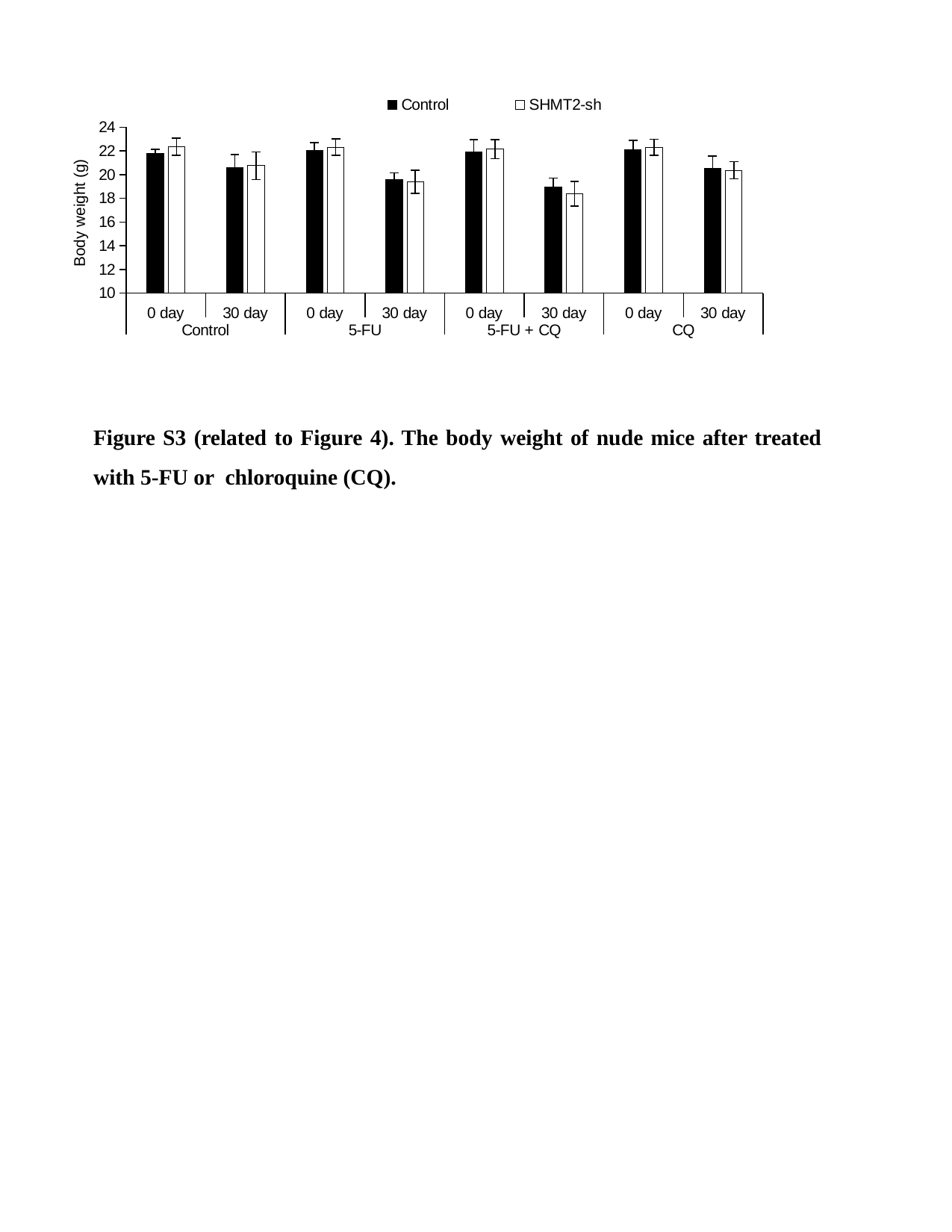

### Chart
| Category | Control | SHMT2-sh |
|---|---|---|
| 0 day | 21.8 | 22.339999999999996 |
| 30 day | 20.6 | 20.759999999999998 |
| 0 day | 22.040000000000003 | 22.32 |
| 30 day | 19.560000000000002 | 19.400000000000002 |
| 0 day | 21.9 | 22.160000000000004 |
| 30 day | 18.96 | 18.380000000000003 |
| 0 day | 22.1 | 22.300000000000004 |
| 30 day | 20.560000000000002 | 20.360000000000003 |Body weight (g)
Figure S3 (related to Figure 4). The body weight of nude mice after treated with 5-FU or chloroquine (CQ).

## Slide 7
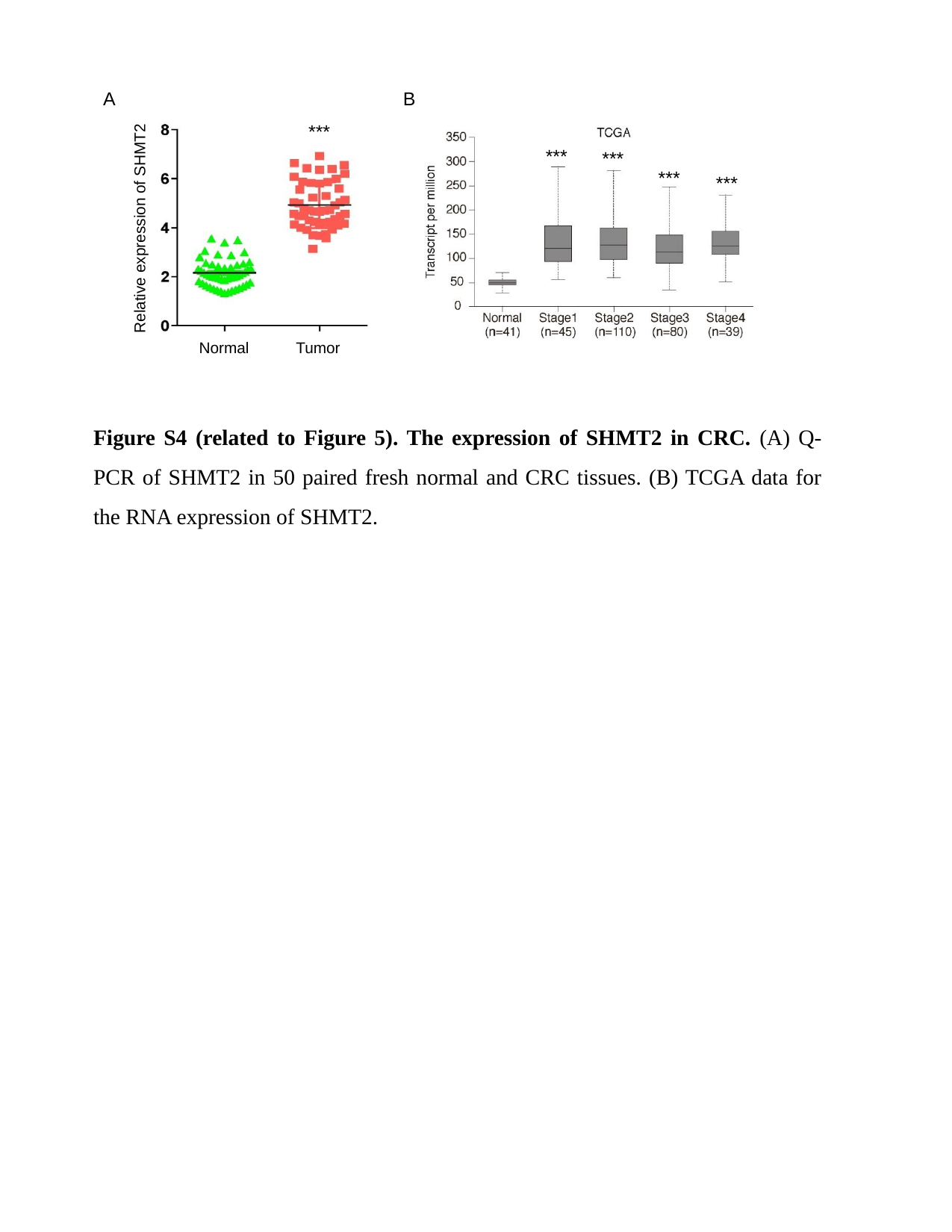

A
B
Relative expression of SHMT2
Normal
Tumor
***
***
***
***
***
Figure S4 (related to Figure 5). The expression of SHMT2 in CRC. (A) Q-PCR of SHMT2 in 50 paired fresh normal and CRC tissues. (B) TCGA data for the RNA expression of SHMT2.

## Slide 8
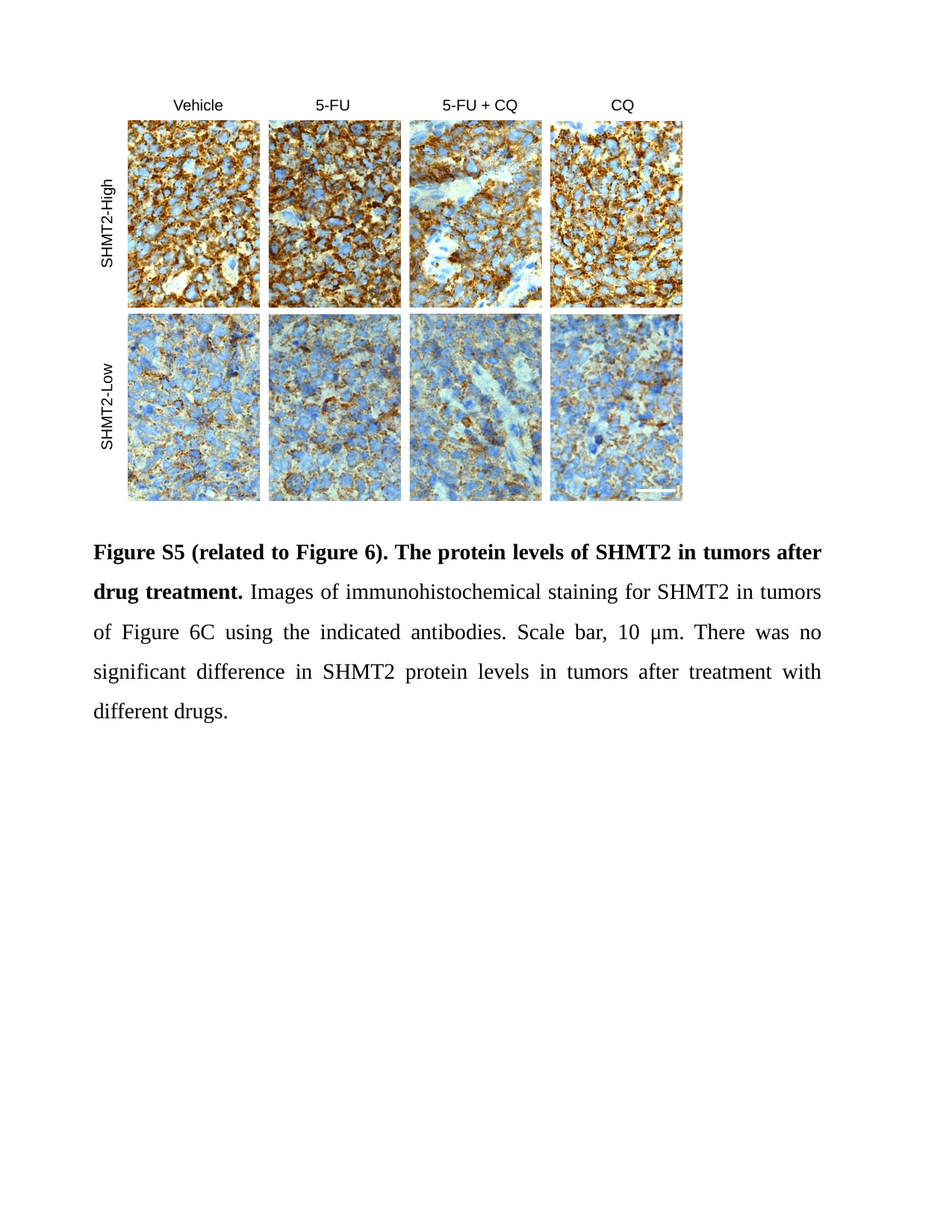

Vehicle
5-FU
5-FU + CQ
CQ
SHMT2-High
SHMT2-Low
Figure S5 (related to Figure 6). The protein levels of SHMT2 in tumors after drug treatment. Images of immunohistochemical staining for SHMT2 in tumors of Figure 6C using the indicated antibodies. Scale bar, 10 μm. There was no significant difference in SHMT2 protein levels in tumors after treatment with different drugs.
