# Supplementary material for: The loss of SHMT2 mediates 5-fluorouracil chemoresistance in colorectal cancer by upregulating autophagy
Source: Oncogene. 2021 May 14;40(23):3974–88. doi: 10.1038/s41388-021-01815-4 (PMC8195740; doi:10.1038/s41388-021-01815-4)
Supplement: Supplementary file 2 — Supplemental Table [file 41388_2021_1815_MOESM2_ESM.docx]

Supplemental Table1.Characteristic of stage II and III patients

|  | SHMT2 low（n=164） | SHMT2 high（n=214） | P value |
| --- | --- | --- | --- |
| Age |  |  | 0.095 |
| ≤65 | 80 (48.8) | 86 (40.2) |  |
| ＞65 | 84 (51.2) | 128 (59.8) |  |
| Gender |  |  | 0.391 |
| Male | 77 (47.0) | 110 (51.4) |  |
| Female | 87 (53.0) | 104 (48.6) |  |
| Location |  |  | ＜0.001 |
| Right | 37 (22.6) | 82 (38.3) |  |
| Left | 61 (37.2) | 62 (29) |  |
| Rectum | 66 (40.2) | 70 (32.7) |  |
| Grade |  |  | 0.409 |
| I | 37 (22.6) | 38 (17.8) |  |
| II | 100 (61.0) | 144 (67.3) |  |
| III | 27 (16.5) | 32 (15.0) |  |
| Tumour size |  |  | 0.709 |
| ＜5cm | 89 (54.3) | 112（52.3） |  |
| ≥5cm | 75 (45.7) | 102（47.7） |  |
| Stage |  |  | 0.990 |
| II | 89 (54.3) | 116（54.2） |  |
| III | 75 (45.7) | 98（45.8） |  |
| Chemo |  |  | 0.930 |
| No | 56（34.15） | 74（34.58） |  |
| Yes | 108 (65.85) | 140 (65.42) |  |

Supplemental table 2.

Cox regression multivariate analysis of disease-free survival in stage II and III patients

|  | Variable | Hazard Ratio | | 95% CI | P |
| --- | --- | --- | --- | --- | --- |
| II-II patients  （n=387） |  |  |  | |  |
|  | Age （＞65 VS. ≤65） | 1.675 | 1.091-2.571 | | 0.018 |
|  | SHMT2 (High VS. Low) | 2.195 | 1.428-3.375 | | ＜0.001 |
|  | TNM stage （III VS. II） | 2.092 | 1.415-3.092 | | ＜0.001 |
|  | Adjuvant CT | 0.267 | 0.180-0.398 | | ＜0.001 |
| Adjuvant Chemo patients （n=248） |  |  |  | |  |
|  | Age（＞65 VS. ≤65） | 1.623 | 0.883-2.982 | | 0.119 |
|  | SHMT2 (High VS. Low) | 0.667 | 0.507-0.879 | | 0.004 |
|  | TNM stage （III VS. II） | 3.153 | 1.755-5.664 | | ＜0.001 |

Supplemental table 3.

Cox regression multivariate analysis of overall survival in stage II and III patients

|  | Variable | Hazard Ratio | 95% CI | P |
| --- | --- | --- | --- | --- |
| II-II patients  （n=387） |  |  |  |  |
|  | Age （＞65 VS. ≤65） | 1.782 | 1.148-2.765 | 0.010 |
|  | SHMT2 (High VS. Low) | 2.037 | 1.322-3.138 | 0.001 |
|  | TNM stage （III VS. II） | 1.858 | 1.251-2.762 | 0.002 |
|  | Adjuvant CT | 0.256 | 0.171-0.384 | ＜0.001 |
| Adjuvant Chemo patients （n=248） |  |  |  |  |
|  | Age（＞65 VS. ≤65） | 1.481 | 0.799-2.746 | 0.212 |
|  | SHMT2 (High VS. Low) | 0.417 | 0.236-0.737 | 0.003 |
|  | TNM stage （III VS. II） | 3.269 | 1.787-5.981 | ＜0.001 |
